# Cerebrospinal ceramides and cognition as a function of striatal asymmetry in early stage of Parkinson's disease

**DOI:** 10.1177/1877718X251319242

**Published:** 2025-03-02

**Authors:** Anthony Nuber-Champier, Philippe Voruz, Ioana Constantin, Alexandre Cionca, Julie A Péron

**Affiliations:** 1 Clinical and Experimental Neuropsychology Laboratory, Faculty of Psychology, University of Geneva, Geneva, Switzerland; 2 Department of Neurosurgery, University Hospitals of Geneva, Geneva, Switzerland; 3 Institute of Bioengineering, Center for Neuroprosthetics, Ecole Polytechnique Fédérale de Lausanne (EPFL), Lausanne, Switzerland; 4 Neurology Division, Geneva University Hospitals, Geneva, Switzerland

**Keywords:** striatal, asymmetry, ceramides, cognition, glucosylceramides, Parkinson's disease

## Abstract

**Background:**

Parkinson's disease (PD) is characterized by heterogeneous clinical phenotypes that may be influenced by the asymmetry of striatal denervation. The alpha-Synuclein Origin site and Connectome (SOC) model proposes that different disease onset patterns—“body first” versus “brain first”—affect symptom development and cognitive decline.

**Objective:**

This study aims to explore the relationship between striatal denervation asymmetry, ceramide metabolism, and cognitive performance in early-stage PD.

**Methods:**

We analyzed data from 329 patients with PD at baseline, categorized by the type of putamen denervation asymmetry predominance at baseline, along with data from 167 healthy controls. We performed generalized linear mixed models introducing ceramides levels and cognitive performance as discriminating factors. Spearman correlations were used to highlight the relationship between cognition and ceramides.

**Results:**

Our findings revealed that patients with asymmetric striatal denervation exhibited higher concentrations of C18:0 ceramides compared to both symmetric patients and healthy controls. Moreover, patients with symmetric denervation demonstrated greater cognitive impairment than those with right or left asymmetry.

**Conclusions:**

The study highlights the importance of striatal denervation asymmetry in influencing ceramide metabolism and cognitive function in early-stage PD. These findings suggest that specific ceramide profiles may serve as metabolic markers to distinguish clinical phenotypes, providing insights into disease progression.

## Plain language summary

Parkinson's disease (PD) manifests as a diversity of symptoms that can vary according to the asymmetry of striatal denervation (a region of the brain). The SOC (alpha-Synuclein Origin site and Connectome) model suggests that the onset of symptoms may begin either in the body (‘body first’) or in the brain (‘brain first’), influencing the progression of symptoms and cognitive decline.

In this study, we analyzed the relationship between striatal denervation asymmetry, ceramide metabolism (types of fat in the brain), and cognitive performance in patients with early-stage PD. We examined data from 329 PD patients, divided according to the type of asymmetry in the striatum, as well as from 167 healthy individuals. Our results show that patients with asymmetric denervation had higher levels of C18:0 ceramides compared to patients with symmetric denervation and healthy controls. In addition, patients with symmetric denervation showed more cognitive impairment. These results suggest the importance of considering striatal denervation asymmetry and that ceramides could be useful metabolic markers for differentiating types of PD and better understanding disease progression.

## Introduction

The alpha-Synuclein Origin site and Connectome (SOC) model offers a novel framework for characterizing the diverse clinical phenotypes observed in people with Parkinson's disease (PwPD).^1^ According to this model, two primary phenotypes can be distinguished based on disease onset: “body first” and “brain first.” Patients exhibiting a “body first” phenotype typically present with symmetrical damage to the nigrostriatal regions, resulting in vegetative symptoms and symmetrical motor impairments. This phenotype often progresses more rapidly toward neurocognitive dementia.^2^ In contrast, those with a “brain first” onset experience asymmetric brain damage, which correlates with asymmetric motor symptoms on the contralateral side of the affected hemisphere and fewer non-motor symptoms. Consequently, this phenotype may experience a slower progression to dementia.

Despite the relevance of the SOC model, the role of metabolic markers in capturing the clinical phenotypes of PwPD remains underexplored, even though altered metabolism has been implicated in disease progression.^3–7^ Glucosylceramides, a type of glucocerebroside, are integral to the metabolism of ceramides (CER) and sphingomyelin (SM) in neuronal tissue.^3,8^ The significance of ceramide metabolism in Parkinson's disease (PD) has been underscored by findings linking GBA1 gene mutations to altered ceramide levels.^9^ However, studies present conflicting results regarding ceramide concentrations in PD patients compared to healthy controls, with some reporting increased levels of C18:0 ceramide and decreased levels of C24:0.^10,11^ Moreover, specific ceramide concentrations in brain grey matter, such as reductions in anterior cingulate cortex regions, have been associated with neurodegeneration in PwPD.^4^

The relationship between cognition and brain ceramides is particularly complex. For instance, one study indicated that higher levels of C16:0, C18:0, C20:0, C22:0, and C24:1 ceramides were linked to greater cognitive impairment in PwPD.^10,12^ Furthermore, specific ceramides like C14:0 and C24:1 have been negatively correlated with verbal memory performance.^12^ Nuanced observations also highlight variations in glucosylceramide (GlcCer) levels in cerebrospinal fluid (CSF), where PD dementia is characterized by an accumulation of GlcCer associated with a decrease in GD1 gangliosides.^13^ Te Vruchte et al.^9^ reported increased GlcCer levels alongside positive correlations between GD1 and global cognitive scores. Longitudinal studies have suggested a connection between the glucosylceramide/sphingomyelin ratio (GlcCer/SM) and accelerated cognitive decline.^14^ Despite the complexity surrounding ceramide metabolism in PD,^15^ the potential interactions between these metabolic markers and cognitive disorders warrant further investigation, particularly in the context of symptom asymmetry.

In this light, the present study has two primary objectives. First, we aim to explore the impact of asymmetric versus symmetric striatal denervation on CSF ceramide accumulation rates and cognition in early-stage PD patients without known genetic mutations. Second, we will investigate the relationship between ceramide accumulation and cognitive performance in relation to striatal asymmetry. Based on the existing literature,^9,10,14^ we hypothesize that patients with asymmetric degeneration in the putamen will exhibit lower levels of CSF glucosylceramides and ceramides, along with less cognitive impairment compared to those with symmetric degeneration. Furthermore, we anticipate finding negative correlations between ceramide and glucosylceramide accumulation and cognitive performance within the symmetric PwPD subgroup.

## Methods

### Power analysis and participant selection

#### Power analysis

The power analysis was set at 1-*β *= 0.80 and *α *= 0.05 and based on the sample of Huh et al.,^14^ split between 114 healthy controls (HC) and 189 idiopathic PwPD. This indicated that we needed to split 85 people into groups to best detect an effect. Considering the asymmetry subgroups based on Fiorenzato et al.,^16^ we estimated the need to include *N *= 70 people by subgroup.

#### Participant criteria

**Figure 1.**
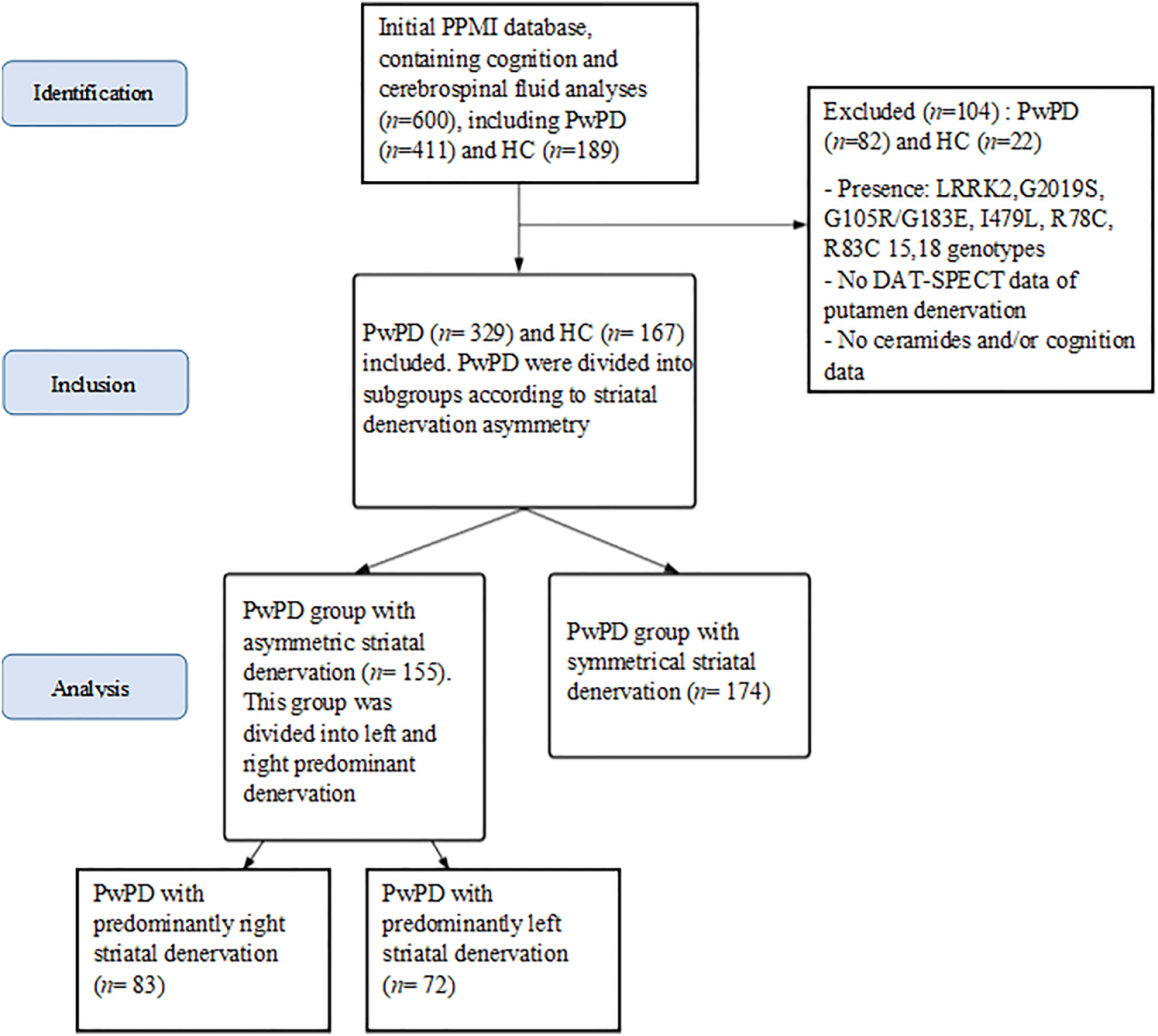
Study flowchart. PwPD with symmetrical striatal denervation accumulating both cognitive and metabolic data represented *n *= 148; PwPDs with asymmetrical striatal denervation accumulating both cognitive and metabolic data represented *n *= 129. PwPD: people with Parkinson's disease; HC: healthy controls.

The dataset used in this article (baseline) was obtained from the Parkinson's Progression Markers Initiative (PPMI) database (www.ppmi-info.org/data).^17^ The global PPMI cohort is composed of male, and female diagnosed patients over 30 years of age, not taking any medication within 6 months of the first baseline visit. Participants had at least two of the following symptoms: resting tremor, bradykinesia and rigidity (must have either resting tremor or bradykinesia). For updated information on this study, see www.ppmi-info.org. Participants with available data for ceramides concentrations, DAT-SPECT analysis, clinical motor evaluation and cognition were retained. Patients with LRRK2, G2019S, G105R/G183E, I479L, R78C, and R83C genotypes^14,18^ and/or no single photon emission computed tomography of the dopamine transporter (DAT-SPECT) data of putamen denervation or other variables of interest (ceramides, cognition) were excluded from the study (see Table 1, Figure 1, and Supplemental Material 1). This initial stage resulted in the selection of 496 individuals, comprising 167 healthy controls and 329 patients with PD (≈6 months from diagnosis to study start, no dopaminergic treatment had been introduced yet).

**Table 1.** Sociodemographic and clinical features of the cohort as a function of striatal (a)symmetry groups.

	HC (*n* = 167)	Left-predominant striatal denervation (n = 72)	Right-predominant striatal denervation (*n*= 83)	Symmetric striatal denervation (*n* = 174)	*p*
Mean age in years (± *SD*)	60.3 ± 11.6	59.4 ± 9.8	59.3 ± 9.96	62.4 ± 8.45	0.057
Men %	64%	51%	65%	64%	0.871
Mean years of education (± SD)	16.2 ± 2.8	15.6 ± 2.4	16.1 ± 2.6	15.7 ± 3.0	0.849
Mean time (months) between diagnosis and baseline measurements (± *SD*)	Na.	6.8 ± 7.11	7.5 ± 7.83	6.62 ± 6.26	0.574
Mean putamen denervation (SBR) right (± SD)	Na.	0.62 ± 0.22 *£	1.13 ± 0.37	0.80 ± 0.32 *	<0.001
Mean putamen denervation (SBR) left (± SD)	Na.	1.15 ± 0.40 *£	0.60 ± 0.22	0.80 ± 0.32 *	<0.001
Handedness (*n* and %) (Left)	22 13.1%	9 12.5%	5 6%	15 8,6%	*ns* *See note*
Handedness (*n* and %) (Right)	135 80%	61 84%	72 86%	157 90%	*ns* *See note*
Handedness (*n* and %) (ambidextrous)	9 5.3%	2 2.7%	6 7.2%	1 0.5%	*ns* *See note*
MDS-UPDRS 3 asymmetry score (Mean ± *SD*)	na	−8.41 ± 5.64 *£	7.76 ± 3.83	−0.19 ± 7.84 *	<0.001
MDS-UPDRS 3 total score (Mean ± *SD*)	na	33.44 ± 11.09 *	29.46 ± 12.39	33.68 ± 13.39 *	0.02
Hoehn and Yahr stage I	na	Stage I: 29 40.2% *	Stage I: 48 57.8%	Stage I: 73 41.9% *	*See above*
Hoehn and Yahr stage II	na	Stage II: 43 59.7% *	Stage II: 35 42.1%	Stage II: 101 58.0% *	*See above*

HC: healthy controls; L: left; na: not applicable; PwPD: people with Parkinson's disease; R: right; SD: standard deviation; SBR: striatal specific binding ratios; MDS-UPDRS: Unified Parkinson's Disease Rating Scale. The differences in striatal denervation are detailed in Supplemental Material 1.

*Difference with Right-predominant striatal denervation.

£Difference with Symmetric striatal denervation.

### Striatal asymmetry calculation and categorization of group patients

In a second stage, we analyzed striatal asymmetry using Kaasinen's calculations of Striatal specific binding ratios (SBR), defining cerebral asymmetry as an SBR difference exceeding 20%, and subsequently categorizing PD patients into groups with striatal asymmetric (*n* = 155) or symmetric denervation (*n *= 174), with the asymmetric group further divided into right-predominant denervation (*n* = 83) and left-predominant denervation (*n* = 72).

As a reminder, according to Kaasinen,^19^ the SBR calculation was carried out by the PPMI SPECT Core Laboratory (Institute for Neurodegenerative Disorders, New Haven, CT) following a process that included iterative reconstruction of raw projection data, site-specific attenuation correction using phantom data, and spatial normalization of images in the Montreal Neurological Institute (MNI) space. Region-of-interest (ROI) analyses were performed using a fixed template, including the right and left caudate, right and left putamen, and occipital cortex, to calculate SBR according to the following formula: ([striatal region count density/occipital count density]-1), enabling assessment of receptor binding in the striatum.

#### Method

As in the Kaasinen^19^ and Fiorenzato et al.^16^ studies, we used the difference in SBR [(right-left putamen SBR)/ (right + left putamen SBR)] to calculate putamen denervation asymmetry. We determined an asymmetry of the putamen when the ratio was superior to 20%.^16^

### Data selection and extraction

In a third stage, we extracted socio-demographical and clinical data, neuropsychological measures along with Glucosylceramides (GlcCer) and ceramides (Cer) data of the selected samples.

#### Participant information

Age, gender, years of education, handedness, time from diagnosis to the start of the study measurements were extracted from the PPMI database for the PwPD and the HC. Movement Disorder Society-Sponsored Revision of the Unified Parkinson's Disease Rating Scale (MDS-UPDRS 3) scores (and an MDS-UPDRS 3 asymmetry index using the lateralized items of the MDS-UPDRS 3 for which we subtracted the left lateralized items from the right lateralized ones) and Hoehn and Yahr stages were extracted for PwPD.

#### Cognitive measures

We used measures of cognition related to verbal episodic memory with the Hopkins verbal learning test (HVLT),^20^ verbal executive function with the Semantic fluency test (SFT),^21^ processing speed with the Symbol digit modalities test (SDMT)^22^ and visuospatial process with the Benton judgment of line orientation (BJLO) test.^23^ We also retained a global measure of cognition with the Montreal cognitive assessment (MoCA).^24^

#### Glucosylceramides (GlcCer) and ceramides (Cer) data

We extracted data for C16:0, C18:0, C20:0, C22:0, C24:1, and C24:0 ceramides (CER), glucosylceramides (GlcCer) and sphingomyelin (SM) (for more details on ceramide extraction, see Huh et al.^14^). Ceramides or glucosylceramides determinations were reported in ng/mL. The galactosylglucosylceramides (GL2) dataset was not extracted due to lack of data.

### Statistical analysis

Kolmogorov Smirnov test revealed the non-parametric nature of the data. Therefore, non-parametric analyzes were performed.

#### Socio-demographical and clinical data

Kruskall-Wallis analyzes were used in order to compare continuous data and χ^2^ to compare categorical data, considering HC, left-predominant striatal denervation, right-predominant striatal denervation and symmetric striatal denervation (see Table 1).

#### Aim 1

In order to investigate metabolic and cognitive differences as a function of striatal denervation, we performed the following generalized linear mixed models (GLMM). We considered the asymmetry of striatal denervation as a fixed effect. These models allow to control for random effects such as inter-individual variability (handedness, SBR denervation of the right and left putamen). We used a GLMM model following gamma regression for each ceramides and each cognitive test. Thus, we were able to consider 4 intra-participant factors: Ceramides (levels 7: C16:0, C18:0, C20:0, C22:0, C23:0, C24:0, C24:1 CER) or Glucosylceramides (levels 7: C16:0, C18:0, C20:0, C22:0, C23:0, C24:0, C24:1 GlcCer) or Sphingomyelin (levels 7: C16:0, C18:0, C20:0, C22:0, C23:0, C24:0, C24:1 SM), or Cognitive performance (4 levels: MoCA, SFT, SDMT, BJLO), and 1 between-participant factor: Group (3 levels: asymmetric striatal denervation, symmetric striatal denervation and HC) or (4 levels: left striatal denervation predominant, right striatal denervation predominant, symmetric striatal denervation and HC).

#### Aim 2

In order to investigate the associations between metabolic markers and cognitive functions, we performed two-way Spearman correlations between significantly discriminating ceramides, glucosylceramides, and cognitive variables on the whole sample and by subgroups.

#### Corrections

The Benjamini-Hochberg false discovery rate (FDR) correction was for each model of analysis. Indeed, studies have shown that Bonferroni is not an adequate correction for positive variables and for a large number of analyzes.^25^ Our decision to perform Benjamini-Hochberg FDR corrections allowed us to avoid type II errors.^26^ We added the significance thresholds for each group comparison in Supplemental Material 3.

#### Software

Analyses were performed with SPSS version 27.0.0.0.a.

### Ethics

The study was conducted in accordance with the Declaration of Helsinki. All participants in the PPMI clinical database had the ability to provide informed consent in accordance with Good Clinical Practice (GCP), the International Conference on Harmonisation (ICH) and local regulations.

## Results

### Sociodemographic and clinical data

We analyzed sociodemographic data at baseline of the disease according to the striatal denervation asymmetry index (i.e., no dopaminergic treatment had been introduced yet). Participants were comparable in age, gender, education, and time from diagnosis to the start of the study measurements (see Table 1 and Supplemental Material 1). The sociodemographic and cognitive analyses were performed on 167 HC, 174 symmetric PwPD, and 155 asymmetric PwPD and the ceramides analyses on 123 HC, 148 symmetric PwPD, and 129 asymmetric PwPD (see Supplemental Material 2).

The proportion of right-handed people was not significantly different between the groups with left-predominant striatal denervation asymmetry and those with right-predominant striatal denervation asymmetry (*p *= 0.72; χ^2 ^= 0.12), the group with left-predominant striatal asymmetric denervation vs. the group with symmetric denervation (*p *= 0.18; χ^2 ^= 1.76) and the group with right-predominant striatal denervation asymmetry vs. the group with symmetrical denervation (*p *= 0.34; χ^2 ^= 0.89). The proportion of left-handed people was not significantly different between the group with left-predominant striatal denervation asymmetry and the group with right-predominant striatal denervation asymmetry (*p *= 0.16; χ^2 ^= 1.97), the group with left-predominant striatal asymmetric denervation vs. the group with symmetric denervation (*p *= 0.34; χ^2 ^= 0.87) and the group with right-predominant striatal denervation asymmetry vs. the group with symmetrical denervation (*p *= 0.46; χ^2 ^= 0.52). The proportion of ambidextrous individuals in the group with right-predominant striatal asymmetric denervation was significantly higher than the proportion of ambidextrous individuals in the group with symmetric denervation (*p *= 0.002; χ^2^* *= 9.31). The proportion of ambidexterity was not significantly different between the group with left-dominant striatal asymmetric denervation and the group with symmetric denervation, nor between the group with left-dominant striatal asymmetric denervation and the group with right-dominant striatal asymmetric denervation (*p *= 0.16; χ^2 ^= 1.92 and *p *= 0.20; χ^2 ^= 1.61). The group with right-predominant striatal denervation asymmetry has a higher proportion of individuals in Hoehn and Yahr stage I than the group with left-predominant striatal denervation asymmetry (*p *= 0.029; χ^2 ^= 4.72) and the group with symmetrical denervation (*p *= 0.017; χ^2 ^= 5.66). The group with left-predominant striatal denervation asymmetry vs. the group with symmetrical denervation does not differ in the proportion of individuals in stage I Hoehn and Yahr (*p *= 0.80; χ^2 ^= 0.059). The group with right-predominant striatal denervation asymmetry has a lower proportion of individuals in Hoehn and Yahr stage II compared to the group with left-predominant striatal denervation asymmetry (*p *= 0.029; χ^2 ^= 4.72) and the group with symmetrical denervation (*p *= 0.017; χ^2 ^= 5.66). The group with left-predominant striatal denervation asymmetry vs. the group with symmetrical denervation does not differ in the proportion of individuals in stage II Hoehn and Yahr (*p *= 0.80; χ^2 ^= 0.05).

### Intergroup differences in CSF ceramides concentration as a function of striatal asymmetry

#### Symmetrical vs. asymmetrical striatal denervation

Considering striatal asymmetry and applying FDR corrections*,* asymmetric PwPD had a higher concentration of C18:0 CER than symmetric PwPD (*t = *−2.26; *p = *0.024), and HC (*t = *2.42; *p = *0.016).

#### Symmetrical vs. left or right predominant striatal denervation

**Figure 2.**
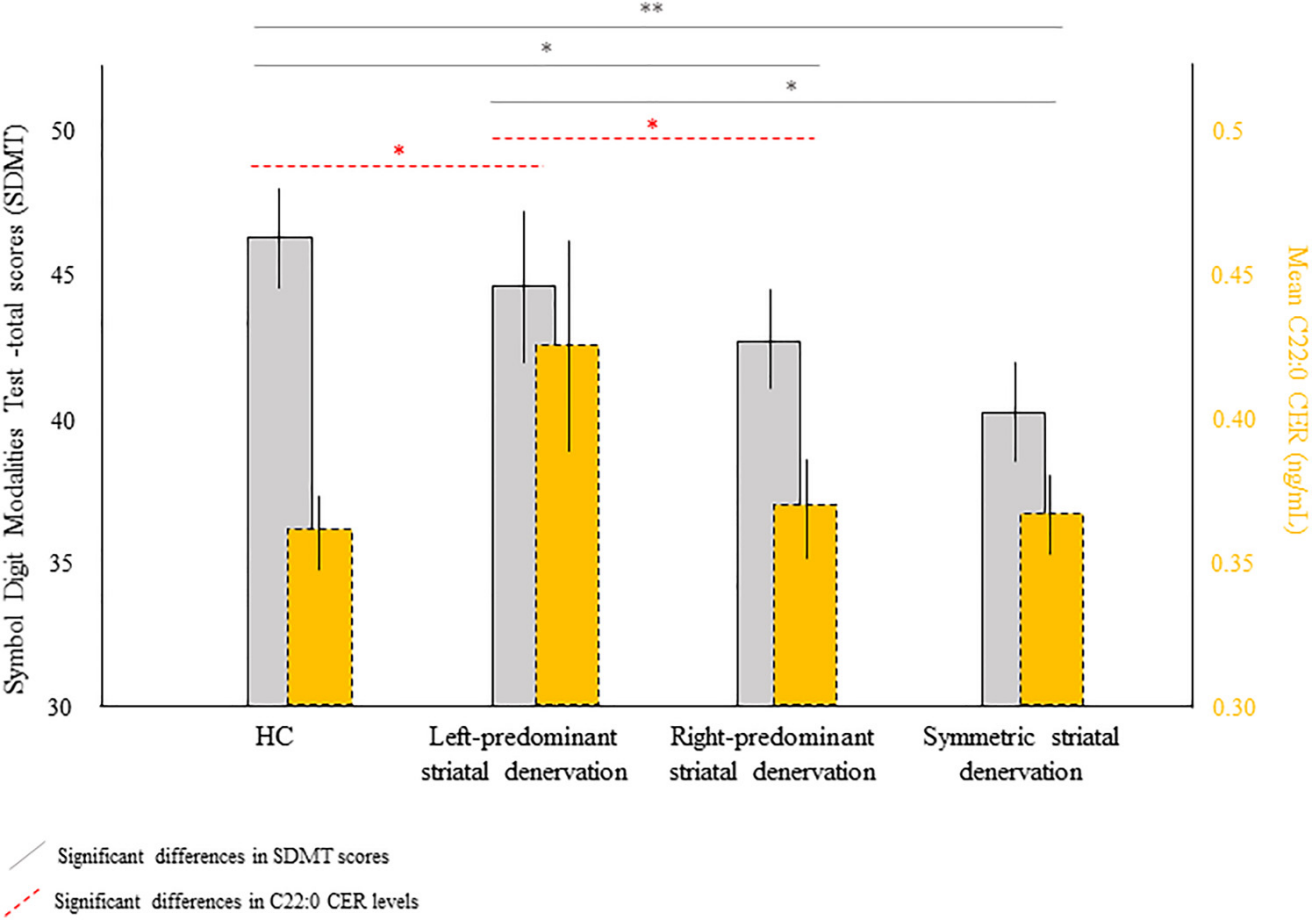
Differences in cognitive performance (SDMT total scores) and ceramide C22:0 as a function of asymmetry of striatal denervation (SBR) subgroups. HC: healthy controls; SBR: striatal specific binding ratios; SDMT: Symbol digit modalities test. 1 standard error is represented by the bars. The asymmetric PwPD corresponds to a difference of at least 20% in the interhemispheric SBR putamen. *FDR corrected, ** < 0.001 FDR corrected.

Moreover, left predominant striatal denervation group had a higher concentration of C22:0 CER than both the right predominant denervation asymmetry group (*t = *−2.60; *p *= 0.010) and HC (*t = *−2.65; *p *= 0.008) (see Figure 2).

All other comparisons did not survive FDR correction.

### Intergroup differences in cognitive performances as a function of striatal asymmetry

#### Symmetrical vs. asymmetrical striatal denervation

Considering striatal asymmetry and applying FDR correction*,* asymmetrical PwPD had better cognitive performances on the total score of symbol digit modalities test (SDMT) (*t = *−3.62; *p < *0.001) and the Montreal cognitive assessment (MoCA) scores (*t = *−2.60; *p = *0.010) as compared to the symmetric group.

#### Symmetrical vs. left or right predominant striatal denervation

Left predominant striatal denervation group had better performances in the SDMT total score as compared to the symmetric group (*t = *−3.45; *p = *0*.*001) (see Figure 2). Right predominant striatal denervation group were also better than the symmetric PwPD group on the global cognitive scale (MoCA) (*t = *−2.62; *p = *0.009).

Unsurprisingly, the HC displayed better performances than the PD group, and subgroups: HC vs. left-predominant striatal denervation asymmetry PwPD for the MoCA (*t *= 3.50; *p < *0.001), HC vs. right-predominant striatal denervation asymmetry PwPD for the SDMT (*t *= 2.73; *p = *0.006) and the MoCA (*t *= 2.64; *p *= 0.009), HC vs. asymmetric PwPD for the SDMT (*t = *2.50; *p *= 0.013) and the MoCA (*t *= 3.74; *p *< 0.001), HC vs. symmetric PwPD for the SDMT (*t *= 6.23; *p < *0.001), MoCA (*t *= 6.48; *p *< 0.001) and HVLT immediate recall (*t *= 4.03; *p *< 0.001).

All other comparisons were not significant after FDR correction (Table 2).

**Table 2.** Ceramides levels and cognitive performances according to striatal denervation asymmetry groups (SBR).

	HC *^ç^ n *= 123 *^#^ n *= 167 (Mean ± *SD*)	Left-predominant striatal denervation asymmetry ^ç^ *n *= 57 *^#^ n *= 72 (Mean ± *SD*)	Right-predominant striatal denervation asymmetry ^ç^ *n *= 72 *^#^ n *= 83 (Mean ± *SD*)	Symmetry of striatal denervation ^ç^ *n *= 148 *^#^ n *= 174 (Mean ± *SD*)	Asymmetry of striatal denervation ^ç^ *n *= 129 *^#^ n *= 155 (Mean ± *SD*)
C16:0 CER ^ç^	0.35 ± 0.13	0.38 ± 0.17	0.35 ± 0.14	0.34 ± 0.16	0.36 ± 0.15
C16:0 GlcCer ^ç^	0.24 ± 0.11	0.29 ± 0.30	0.23 ± 0.10	0.24 ± 0.14	0.26 ± 0.22
C16:0 SM ^ç^	188.35 ± 68.15	196.49 ± 103.24	178.88 ± 80.61	183.90 ± 73.57	186.66 ± 91.35
C18:0 CER ^ç^	2.21 ± 0.72	2.27 ± 0.74	2.17 ± 0.76	2.03 ± 0.77 *	2.21 ± 0.75 ^!^
C18:0 GlcCer ^ç^	0.61 ± 0.28	0.66 ± 0.33	0.59 ± 0.23	0.58 ± 0.23	0.62 ± 0.28
C18:0 SM ^ç^	200.88 ± 66.95	204.42 ± 100.08	190.57 ± 73.21	187.85 ± 66.57	196.70 ± 86.04
C20:0 CER ^ç^	0.29 ± 0.10	0.32 ± 0.14	0.29 ± 0.12	0.28 ± 0.12	0.31 ± 0.13
C20:0 GlcCer ^ç^	0.04 ± 0.10	0.05 ± 0.13	0.04 ± 0.10	0.04 ± 0.11	0.05 ± 0.11
C20:0 SM ^ç^	63.00 ± 21.29	67.10 ± 30.86	60.75 ± 23.41	64.49 ± 29.84	63.55 ± 27.03
C22:0 CER ^ç^	0.36 ± 0.14 ^£^	0.42 ± 0.28	0.37 ± 0.14 ^£^	0.36 ± 0.16	0.39 ± 0.21
C22:0 GlcCer ^ç^	0.48 ± 0.39	0.70 ± 0.70	0.49 ± 0.42	0.54 ± 0.43	0.58 ± 0.56
C22:0 SM ^ç^	63.23 ± 23.01	69.02 ± 33.12	63.50 ± 28.92	66.35 ± 28.24	65.94 ± 30.84
C23:0 CER ^ç^	0.05 ± 0.12	0.13 ± 0.25	0.07 ± 0.13	0.09 ± 0.15	0.10 ± 0.20
C23:0 GlcCer ^ç^	0.36 ± 0.28	0.48 ± 0.37	0.38 ± 0.30	0.41 ± 0.32	0.42 ± 0.33
C23:0 SM ^ç^	17.03 ± 8.67	18.82 ± 11.29	16.43 ± 10.96	18.21 ± 10.23	17.49 ± 11.13
C24:0 CER ^ç^	0.54 ± 0.24	0.65 ± 0.61	0.54 ± 0.28	0.60 ± 0.42	0.59 ± 0.46
C24:0 GlcCer ^ç^	1.81 ± 1.08	2.25 ± 1.62	1.87 ± 1.13	2.00 ± 1.35	2.04 ± 1.38
C24:0 SM ^ç^	25.62 ± 15.21	27.47 ± 17.34	25.04 ± 16.73	26.23 ± 16.95	26.11 ± 16.98
C24:1 CER ^ç^	0.72 ± 0.25	0.83 ± 0.48	0.74 ± 0.35	0.74 ± 0.30	0.78 ± 0.41
C24:1 GlcCer ^ç^	1.82 ± 1.17	2.17 ± 1.87	1.78 ± 1.06	1.99 ± 1.48	1.95 ± 1.48
C24:1 SM ^ç^	50.65 ± 17.67	54.06 ± 24.13	50.56 ± 22.23	53.48 ± 22.03	52.11 ± 23.07
HVLT imm.*^#^*	26.23 ± 4.65	24.64 ± 4.60	25.34 ± 4.57	24.11 ± 5.16*	25.01 ± 4.58
HVLT ret. *^#^*	0.90 ± 0.18	0.84 ± 0.17	0.86 ± 0.18	0.84 ± 0.20	0.85 ± 0.18
SDMT *^#^*	47.24 ± 10.76	44.86 ± 9.40 ^!^	42.93 ± 8*	40.09 ± 10.50*	43.83 ± 8.70 ^!*^
SFT *^#^*	52.26 ± 11.32	48.40 ± 10.84	51.82 ± 12.93	48.41 ± 11.70	50.23 ± 12.08
BJLO *^#^*	13.17 ± 1.96	13.04 ± 1.99	12.62 ± 2.25	12.66 ± 2.22	12.81 ± 2.14
MoCA *^#^*	28.22 ± 1.15	27.3 ± 2.50*	27.53 ± 2.03 ^!*^	26.80 ± 2.31*	27.42 ± 2.25 ^!*^

CER: ceramides; HC: Healthy controls; HVLT imm.: Hopkins verbal learning test immediate recall; HVLT ret: Hopkins verbal learning test retention; MoCA: Montreal cognitive assessment; PwPD: people with Parkinson disease; SBR: striatal specific binding ratios; SDMT: Symbol digit modalities test; SFT: Semantic fluency total score; SM: sphingomyelin; SPD: Symmetric putamen denervation.

The number of people for the ceramides data (*n* annotated ç) and neuropsychological data (*n* annotated #). FDR-corrected (for detailed see Supplemental material 3).

* Significance with HC; ^£^ Significance with the Left-predominant striatal denervation asymmetry PwPD; ^!^ Significance with the symmetric group.

### Relation between CSF ceramides and cognition as a function of striatal denervation asymmetry subgroups

#### Symmetrical and asymmetrical striatal denervation subgroup

Interestingly, we observed almost uniquely positive correlations with cognition (SDMT and HVLT) for ceramides (C18:0 CER and C22:0 CER), and mainly negative correlations with cognition (SDMT and SFT) for glucosylceramides (C18:0 GlcCer and C22:0 GlcCer). More specifically, we observed negative correlations between C22:0 GlcCer and SDMT for the symmetrical group (*r = *−0.20; *p = *0.015) and the asymmetrical group (*r = *−0.25; *p = *0.004). There were also significant positive correlations after correction only for the asymmetric group between C18:0 CER, C22:0 CER, and SDMT (*r = *0.21; *p = *0.017 and *r = *0.20; *p = *0.021) (see Supplemental Material 4).

#### Left or right predominant striatal denervation subgroup

We observed the same patterns of results when analyzes were performed on the whole sample, or within subgroups, except for the left predominant striatal asymmetric group where no correlation was found (see Supplemental Material 4).

Regarding the association between ceramides, glucosylceramides and cognitive performance (SFT, SDMT, HVLT, BJLO, and MoCA), we found a positive correlation between C22:0 CER and SDMT (*r = *0.25; *p = *0.030) and a negative correlation between C22:0 GlcCer and SFT (*r = *−0.31; *p = *0.008) in the right predominant striatal denervation subgroup.

## Discussion

This study aimed to investigate the impact of asymmetric striatal denervation on ceramide accumulation rates in CSF and cognitive performance in early-stage PD patients. Our findings indicate that patients with asymmetric striatal denervation exhibit distinct patterns of ceramide levels and cognitive function compared to those with symmetric denervation.

We found that patients with asymmetric striatal denervation, particularly those with left-predominant degeneration, had higher concentrations of specific ceramides, such as C18:0 and C22:0, compared to both the symmetric PD and healthy control (HC) groups. This suggests that asymmetric striatal denervation is associated with altered lipid metabolism, which may play a role in the cognitive profiles observed. Interestingly, while elevated ceramide levels, particularly C18:0, correlated positively with cognition in the asymmetric group, C22:0 ceramides showed no such association, indicating that their role might be more complex. These results align with the SOC model,^1^ which posits that symmetric dopaminergic denervation reflects a higher neuropathological burden and correlates with accelerated cognitive decline. However, our findings suggest that it would be pertinent for the SOC model to further consider the distinctions between left and right striatal asymmetry, as our results demonstrate that cognitive and metabolic outcomes vary significantly based on the side of degeneration.

The cognitive performance of patients with asymmetric striatal denervation was notably better than that of the symmetric group, as assessed by the SDMT and MoCA. This finding aligns with the notion that asymmetrical patients may develop compensatory mechanisms to cope with cognitive deficits, as highlighted by the observed differences in cognitive performance across subgroups.^1^ Specifically, right-predominant asymmetric denervation was associated with positive correlations between ceramide concentrations and cognitive performance, while the symmetrical group exhibited the poorest cognitive outcomes.

Curiously, we also observed that glucosylceramide accumulation was consistently negatively correlated with cognitive performance across all groups. This suggests a detrimental role of glucosylceramides in cognitive processes, contrasting with the potentially protective or compensatory effects of certain ceramides, particularly in patients with left-predominant denervation.^10,27^ Our findings raise intriguing questions regarding the metabolic adaptations in patients with different striatal denervation patterns and the implications for understanding cognitive decline in PD.^28^

Our results also support the view that ceramide metabolism could be a future biomarker in PD. Indeed, ceramide mechanisms can influence apoptosis, inflammatory phenomena and synaptic signaling. In this sense, variations in ceramides can affect cell membranes and lipid organization, impacting neuronal activity and cognition.^10,27^ More specifically, ceramide mechanisms can modulate glutamatergic N-methyl-D-aspartate (NMDA) and α-amino-3-hydroxy-5-methyl-4-isoxazolepropionate (AMPA) receptors, essential for long-term potentiation and synaptic transmission.^10,27^ These receptors facilitate Na^+^ and Ca^2+^ transport, in turn triggering intracellular cascades that strengthen synaptic connections, influencing memory consolidation and complex attentional processes. In this way, ceramide variations can disrupt the balance between long-term potentiation and long-term depression, or alter the ionic balance, impairing cognition. With specific regard to PD, it would appear that deregulation of ceramide metabolism, by increasing ceramide levels, may promote alpha-synuclein aggregation and amplify inflammatory responses, leading to cognitive impairment.^10,27^

While our results contribute valuable insights into the relationship between striatal denervation, ceramide metabolism, and cognition in PD, there are limitations to consider. First, while we accounted for genetic factors known to influence the development of Parkinson's disease in patients (PwPD), there may still be other genetic components that could affect the outcomes. Additionally, we found that the absolute values of putamen denervation were higher in patients with symmetric denervation compared to those with asymmetric denervation, indicating a potential neurodegenerative distinction between the two groups. Although we controlled for these denervation differences in our analyzes, a deeper understanding of their implications is necessary. Finally, our assessment focused on executive function, memory, processing speed, and overall cognitive processes based on the available data; however, future research should explore these relationships in relation to other cognitive functions such as emotion recognition or social cognition.

## Conclusion

In conclusion, this study underscores the importance of considering striatal denervation asymmetry when evaluating biomarkers and cognitive performance in PD patients. Our results suggest that specific ceramides may serve as potential markers for disease progression and cognitive outcomes. Understanding the nuances of lipid metabolism in relation to PD could yield important insights into the pathophysiology of cognitive decline and inform therapeutic strategies aimed at preserving cognitive function in this population.

## Supplemental Material

sj-docx-1-pkn-10.1177_1877718X251319242 - Supplemental material for Cerebrospinal ceramides and cognition as a function of striatal asymmetry in early stage of Parkinson's disease Supplemental material, sj-docx-1-pkn-10.1177_1877718X251319242 for Cerebrospinal ceramides and cognition as a function of striatal asymmetry in early stage of Parkinson's disease by Anthony Nuber-Champier, Philippe Voruz, Ioana Constantin, Alexandre Cionca and Julie A Péron in Journal of Parkinson's Disease
