# Supplementary material for: Cerebrospinal ceramides and cognition as a function of striatal asymmetry in early stage of Parkinson's disease
Source: J Parkinsons Dis. 2025 Mar 2:1877718X251319242. Online ahead of print. doi: 10.1177/1877718X251319242 (PMC13347440; doi:10.1177/1877718X251319242)
Supplement: Supplementary file 1 [file sj-docx-1-pkn-10.1177_1877718X251319242.docx]

**Supplemental Material**

**Cerebrospinal ceramides and cognition as a function of striatal asymmetry in early stage of Parkinson's disease**

# Supplemental Material 1

**Mean denervation of the right and left putamen for each subgroup as a function of striatal asymmetry**

PwPD: people with Parkinson disease; SBR: striatal specific binding ratios; 1 standard error is represented by the bars. The difference between the groups marked ** is significant after FDR correction (all comparisons involved *p*<0.001). Asymmetric degeneration corresponds to a difference of at least 20% in the interhemispheric SBR putamen. Here we observed differences in the sense of asymmetry, with left asymmetric PwPD having greater denervation of the left putamen than symmetric or right asymmetric PwPD. Similarly, right asymmetric PwPD have greater denervation of the putamen on the right than other groups. However, we observe that symmetrical PwPD have a higher baseline denervation. Thus, the right denervation of left asymmetric PwPD is significantly lower than symmetric PwPD, as is the left denervation of right asymmetric PwPD compared to symmetric PwPD.

**Supplemental Material 2**

**Sociodemographic and clinical features of the cohort as a function of striatal (a)symmetry groups for patients with ceramides data**

|  | **HC**  **(n=123)** | **Left-predominant striatal denervation**  **(n=57)** | **Right-predominant striatal denervation**  **(n=72)** | **Symmetric striatal denervation**  **(n=148)** | **p** |
| --- | --- | --- | --- | --- | --- |
| Mean age in years (± *SD*) | 61.2 ± 11.2 | 59.7 ± 9.3 | 59.6 ± 9.6 | 62.5 ± 8.5 | 0.10 |
| Men % | 67% | 61% | 65% | 62% | 0.75 |
| Mean years of education (± SD) | 16.4 ± 2.8 | 16.0 ± 2.1 | 16.1 ± 2.6 | 15.7 ± 3.1 | 0.21 |
| Mean time (months) between diagnosis and baseline measurements (± *SD*) | na | 5.65 ± 5.93 | 7.33 ± 7.60 | 6.57 ± 6.24 | 0.18 |
| Mean putamen denervation (SBR):  right (± SD) | na | 0.62 ± 0.19 *£ | 1.13 ± 0.38 | 0.79 ± 0.33 * | <0.001 |
| Mean putamen denervation (SBR):  left (± SD) | na | 1.15 ± 0.38 *£ | 0.60 ± 0.23 | 0.80 ± 0.33 * | <0.001 |
| Handedness (*n* and %) (Left) | 18  14.6% | 7  12.2% | 4  5.5% | 15  10.1% | *ns*  *See note* |
| Handedness (*n* and %) (Right) | 100  81% | 48  84.2% | 63  87.5% | 132  89% | *ns*  *See note* |
| Handedness (*n* and %) (ambidextrous) | 5  4% | 2  3.5% | 5  6.9% | 1  0.6% | *ns*  *See note* |
| MDS-UPDRS 3 asymmetry score (Mean ± *SD*) | na | -8.14 ± 5.95 *£ | 7.46 ± 3.73 | -0.11 ± 7.84 ***** | <0.001 |
| MDS-UPDRS 3 total score (Mean ± *SD*) | na | 34.35 ± 10.61 * | 29.19 ± 12.15 | 33.49 ± 13.33 * | <0.001 |
| Hoehn and Yahr stage I | na | Stage I: 23  40.3% ***** | Stage I: 47  65.2% | Stage I: 64  43.2%***** | *See note* |
| Hoehn and Yahr stage II | na | Stage II: 34  59.6% ***** | Stage II: 25  34.7% | Stage II: 84  56.7%***** | *See note* |

HC: healthy controls; L: left; na: not applicable; PwPD: People with Parkinson’s disease; R: right; SD: standard deviation; SBR: striatal specific binding ratios; MDS-UPDRS: Unified Parkinson’s Disease Rating Scale.

* Difference with Right-predominant striatal denervation

£ Difference with Symmetric striatal denervation

The proportion of right-handed people was not significantly different between the groups with left-predominant striatal denervation asymmetry and those with right-predominant striatal denervation asymmetry (*p*=0.59; χ^2^=0.28), the group with left-predominant striatal asymmetric denervation vs. the group with symmetric denervation (*p*=0.35; χ^2^=0.87) and the group with right-predominant striatal denervation asymmetry vs the group with symmetrical denervation (*p*=0.74; χ^2^=0.10). The proportion of left-handed people was not significantly different between the group with left-predominant striatal denervation asymmetry and the group with right-predominant striatal denervation asymmetry (*p*=0.17; χ^2^=1.83), the group with left-predominant striatal asymmetric denervation vs. the group with symmetric denervation (*p*=0.66; χ^2^=0.18) and the group with right-predominant striatal denervation asymmetry vs. the group with symmetrical denervation (*p*=0.25; χ^2^=1.29). The proportion of ambidextrous individuals in the group with right-predominant striatal asymmetric denervation was significantly higher than the proportion of ambidextrous individuals in the group with symmetric denervation (*p*=0.006; χ^2^=7.38). The proportion of ambidexterity was not significantly different between the group with left-dominant striatal asymmetric denervation and the group with symmetric denervation, nor between the group with left-dominant striatal asymmetric denervation and the group with right-dominant striatal asymmetric denervation (*p*=0.11; χ^2^=2.48 and *p*=0.39; χ^2^=0.71). The group with right-predominant striatal denervation asymmetry has a higher proportion of individuals in Hoehn and Yahr stage I than the group with left-predominant striatal denervation asymmetry (*p*=0.005; χ^2^=7.88) and the group with symmetrical denervation (*p*=0.002; χ^2^=9.33). The group with left-predominant striatal denervation asymmetry vs. the group with symmetrical denervation does not differ in the proportion of individuals in stage I Hoehn and Yahr (*p*=0.70; χ^2^=0.14). The group with right-predominant striatal denervation asymmetry has a lower proportion of individuals in Hoehn and Yahr stage II compared to the group with left-predominant striatal denervation asymmetry (*p*=0.005; χ^2^=7.88) and the group with symmetrical denervation (*p*=0.002; χ^2^=9.33). The group with left-predominant striatal denervation asymmetry vs. the group with symmetrical denervation does not differ in the proportion of individuals in stage II Hoehn and Yahr (*p*=0.70; χ^2^=0.14).

**Supplemental Material 3**

*FDR correction for group differences in ceramides concentrations and cognition with respect to striatal asymmetry*

*Corrected values (FDR) for ceramides differences:*

HC vs. Left-predominant striatal denervation asymmetry PwPD (*p* < 0.05).

HC vs. symmetric PwPD (*p* < 0.05).

Left-predominant striatal denervation asymmetry vs. Right-predominant striatal denervation asymmetry PwPD (*p* < 0.05).

Asymmetric vs. symmetric PwPD (*p* < 0.05).

*Corrected values (FDR) for cognitive differences:*

HC vs. Left-predominant striatal denervation asymmetry PwPD (*p* < 0.05).

HC vs. Right-predominant striatal denervation asymmetry PwPD (*p* < 0.025).

HC vs. symmetric PwPD (*p* < 0.016); HC vs. asymmetric PwPD (*p* < 0.025).

Asymmetric vs. symmetric PwPD (*p* < 0.025).

Right-predominant striatal denervation asymmetry vs. symmetric PwPD (*p* < 0.05).

Left-predominant striatal denervation asymmetry vs. symmetric PwPD (*p* < 0.05).

# Supplemental Material 4

**Correlations between ceramides, glucosylceramides, and cognition**

*Relationship between ceramides, glucosylceramides, and cognition in the whole cohort and by subgroups (symmetrical and asymmetrical striatal denervation (SBR)).*

| **Ceramides/ Glucosylceramides** | **Cognitive tests** | **Whole group**  **n=277** | **p** | **Symmetry of striatal denervation**  **n=148** | **p** | **Asymmetry of striatal denervation**  **n=129** | **p** |
| --- | --- | --- | --- | --- | --- | --- | --- |
| C22:0 GlcCer | SDMT | r=-0.06 | 0.23 | r=-0.20 | **0.015*** | r=-0.006 | 0.94 |
| C18:0 GlcCer | SDMT | r=-0.09 | 0.07 | r=-0.14 | 0.072 | r=-0.08 | 0.33 |
| C22:0 GlcCer | SFT | r=-0.18 | <0.**001*** | r=-0.87 | 0.29 | r=-0.25 | **0.004*** |
| C22:0 CER | SFT | r=-0.08 | 0.09 | r=-0.10 | 0.20 | r=-0.06 | 0.46 |
| C18:0 CER | SFT | r=0.09 | 0.06 | r=0.12 | 0.12 | r=0.05 | 0.57 |
| C18:0 GlcCer | SFT | r=-0.04 | 0.35 | r=-0.17 | 0.83 | r=-0.06 | 0.49 |
| C18:0 CER | SDMT | r=0.16 | 0.**002*** | r=0.15 | 0.07 | r=0.21 | **0.017*** |
| C22:0 CER | SDMT | r=0.05 | 0.25 | r=-0.66 | 0.43 | r=0.20 | **0.021** |
| C18:0 CER | HVLT ret. | r=0.13 | 0.**011*** | r=0.11 | 0.15 | r=0.15 | 0.089 |
| C22:0 CER | HVLT ret. | r=-0.01 | 0.79 | r=-0.02 | 0.76 | r=0.03 | 0.65 |
| C18:0 GlcCer | HVLT ret. | r=0.01 | 0.81 | r=-0.03 | 0.68 | r=0.07 | 0.43 |
| C22:0 GlcCer | HVLT ret. | r=-0.06 | 0.17 | r=-0.06 | 0.46 | r=-0.04 | 0.65 |
| C18:0 CER | BJLO | r=0.05 | 0.31 | r=0.07 | 0.35 | r=-0.03 | 0.70 |
| C22:0 CER | BJLO | r=-0.04 | 0.40 | r=-0.07 | 0.39 | r=-0.01 | 0.84 |
| C18:0 GlcCer | BJLO | r=0.00 | 0.89 | r=-0.07 | 0.37 | r=0.02 | 0.82 |
| C22:0 GlcCer | BJLO | r=0.27 | 0.58 | r=-0.06 | 0.46 | r=0.11 | 0.21 |
| C18:0 CER | MoCA | r=0.05 | 0.28 | r=0.00 | 0.94 | r=0.00 | 0.94 |
| C22:0 CER | MoCA | r=-0.01 | 0.80 | r=-0.09 | 0.24 | r=0.06 | 0.46 |
| C18:0 GlcCer | MoCA | r=-0.06 | 0.22 | r=-0.01 | 0.90 | r=-0.14 | 0.11 |
| C22:0 GlcCer | MoCA | r=-0.03 | 0.43 | r=0.07 | 0.35 | r=-0.07 | 0.38 |

BJLO: Benton judgment of line orientation; CER: ceramides; GlcCer: glucosylceramides; HVLT ret: Hopkins verbal learning test retention; SDMT: Symbol digit modalities test; SFT: Semantic fluency test. Asymmetric putamen denervation (SBR) grouping the right and left striatal denervation predominance.

*FDR corrected

| **Ceramides/ Glucosylceramides** | **Cognitive tests** | **Left-predominant striatal denervation n=57** | **p** | **Right-predominant striatal denervation n=72** | **p** |
| --- | --- | --- | --- | --- | --- |
| C22:0 GlcCer | SDMT | r=-0.10 | 0.43 | r=0.05 | 0.62 |
| C18:0 GlcCer | SDMT | r=-0.07 | 0.59 | r=-0.11 | 0.33 |
| C22:0 GlcCer | SFT | r=-0,07 | 0.60 | r=-0.31 | **0.008*** |
| C22:0 CER | SFT | r=-0.05 | 0.70 | r=-0.04 | 0.69 |
| C18:0 CER | SFT | r=0.08 | 0.52 | r=-0.00 | 0.98 |
| C18:0 GlcCer | SFT | r=0.08 | 0.52 | r=-0.14 | 0.23 |
| C18:0 CER | SDMT | r=0.14 | 0.28 | r=0.22 | 0.053 |
| C22:0 CER | SDMT | r=0.12 | 0.34 | r=0.25 | **0.030*** |
| C18:0 CER | HVLT ret. | r=0.08 | 0.52 | r=0.18 | 0.11 |
| C22:0 CER | HVLT ret. | r=-0.17 | 0.19 | r=0.20 | 0.08 |
| C18:0 GlcCer | HVLT ret. | r=0.08 | 0.52 | r=0.07 | 0.55 |
| C22:0 GlcCer | HVLT ret. | r=-0.17 | 0.19 | r=0.06 | 0.59 |
| C18:0 CER | BJLO | r=0.01 | 0.92 | r=-0.09 | 0.44 |
| C22:0 CER | BJLO | r=0.02 | 0.82 | r=-0.08 | 0.45 |
| C18:0 GlcCer | BJLO | r=0.07 | 0.59 | r=-0.04 | 0.69 |
| C22:0 GlcCer | BJLO | r=0.14 | 0.29 | r=0.06 | 0.60 |
| C18:0 CER | MoCA | r=-0.05 | 0.69 | r=0.03 | 0.79 |
| C22:0 CER | MoCA | r=0.15 | 0.26 | r=0.04 | 0.70 |
| C18:0 GlcCer | MoCA | r=-0.02 | 0.84 | r=-0.19 | 0.10 |
| C22:0 GlcCer | MoCA | r=0.02 | 0.86 | r=-0.12 | 0.30 |

BJLO: Benton judgment of line orientation; CER: ceramides; GlcCer: glucosylceramides; HVLT ret: Hopkins verbal learning test retention; SDMT: Symbol digit modalities test; SFT: Semantic fluency test. Asymmetric putamen denervation (SBR) grouping the right and left striatal denervation predominance.

*FDR corrected
